
JOURNAL INFORMATION
==============================
Journal ID: Wirtschaftsdienst

Wirtschaftsdienst

EISSN: 1613-978X

ARTICLE INFORMATION
==============================
PMCID: PMC8933200
DOI: 10.1007/s10273-022-3126-3
Article ID: 3126
Article version: 1
Subjects: Zeitgespräch

Verschuldung von Verbraucher:innen in Deutschland: Stand und Entwicklung

Consumer Debt in Germany: Status and Development

Peters Sally sally.peters@iff-hamburg.de

grid.506341.5 0000 0001 1089 0930 institut für finanzdienstleistungen e.V. (iff), Grindelallee 100, 20146 Hamburg, Deutschland
Electronic publication date: 2022 Mar 19
Print publication date: 2022
Volume: 102
Issue: 3
First page: 162
Last page: 165
Copyright: © Der/die Autor:in 2022
License: Open Access: Dieser Artikel wird unter der Creative Commons Namensnennung 4.0 International Lizenz veröffentlicht (https://creativecommons.org/licenses/by/4.0/deed.de).
Open Access wird durch die ZBW — Leibniz-Informationszentrum Wirtschaft gefördert.
License URL: https://creativecommons.org/licenses/by/4.0/

Keywords: G51, G53
issue-copyright-statement: © The Author(s) 2022

==============================

Die Lage von Personen in finanziell schwierigen Situationen wird maßgeblich durch die politischen und strukturellen Rahmenbedingungen in Deutschland geprägt. Durch die Gesetzgebung werden unter anderem Rechte und Pflichten der Darlehensnehmer:innen und Gläubiger:innen geregelt; sie ist somit zentral für die Überschuldungsprävention, aber auch für die Rehabilitation überschuldeter Personen.

2021 wurde vor allem durch einen Meilenstein geprägt: die Verkürzung des Insolvenzverfahrens, die am 1.10.2020 in Kraft trat. Justizministerin Lambrecht sagte dazu im März auf dem Deutschen Insolvenzrechtstag, dass die „Restschuldbefreiung nach drei Jahren für Verbraucher:innen ein Gebot der Gerechtigkeit war“ (Lambrecht, 2021, 9).

Mit großer Spannung wurde der Vorschlag für eine Richtlinie des Europäischen Parlaments und des Rates über Verbraucherkredite (CCD-E) erwartet, die im Frühjahr 2022 verabschiedet werden soll. Die Anpassung der Verbraucherkreditrichtlinie an aktuelle Entwicklungen auf dem Kreditmarkt ist notwendig, um auf damit einhergehende Risiken zu reagieren und ist somit zu begrüßen. Das Vorhaben, Plattformen für Peer-to-Peer-Kredite, Minikredite (Nettodarlehensbetrag geringer als 200 Euro), Kurzzeitkredite (Laufzeit kürzer als drei Monate) sowie zinslose Kredite dem Anwendungsbereich der durch die Richtlinie formulierten Regelungen des Verbraucherkreditrechts zu unterstellen, ist zeitgemäß und dringend notwendig. Seit langem häuften sich Forderungen, dass diese Kreditformen verstärkte verbraucherschutzrechtliche Regulierung benötigen. Bisher waren sie vom Verbraucherdarlehensrecht ausgenommen (unter anderem Roggemann et al., 2021a).

Als positiv und dringend notwendig zu bewerten, ist zudem auch die vorgesehene Verpflichtung, dass Mitgliedstaaten finanzielle Allgemeinbildung fördern und die Verfügarbeit von Schuldnerberatung verbessern sollen. Verbesserungswürdig aus Verbrauchersicht und damit auch im Sinne der Überschuldungsprävention ist hingegen, dass beim Thema Finanzbildung kein Schwerpunkt auf die Kenntnis der eigenen Verbraucherrechte und deren Durchsetzung gelegt wird. Zudem legt der Richtlinienentwurf einen Fokus auf Informationsrechte (z. B. beim Thema Überziehungskredite) anstatt schon – wie seit Jahren gefordert – für einen Zinsdeckel für Dispositionskredite zu plädieren (unter anderem Klinger, 2021).

Auch im Bereich des Bankkontos gab es diverse Diskussionspunkte, die aber bisher nur zu wenigen Veränderungen führten. Für vulnerable Gruppen ist das Bankkonto ein entscheidendes Instrument. Zum einen ist es Voraussetzung für die Teilhabe am digitalen Zahlungsverkehr, zum anderen stellt eine (meist) mögliche Überziehung des Kontos einen niedrigschwelligen Zugang zu Krediten dar, um in Zeiten von finanziellen Krisen Liquiditätsengpässe auszugleichen. Dass der damit einhergehende Dispositionszins mit bis zu zweistelligen Dispozinsen zu hoch ist, zeigen insbesondere zwei Studien der Finanzwende (Bürgerbewegung Finanzwende, 2020a, 2020b). Es sind insbesondere Personen mit einem geringen Einkommen (z. B. Arbeitslose, Alleinerziehende, Paare mit Kindern und Selbständige), die den Dispositionskredit nutzen (Dick et al., 2012). Mit dem sogenannten P-Kontofortentwicklungsgesetz gibt es seit dem 1.12.2021 immerhin erhebliche Neuregelungen beim Pfändungsschutzkonto. Die Neuregelungen bringen verschiedene Verbesserungen für Überschuldete mit sich, die den Alltag im Zahlungsverkehr deutlich erleichtern. Auch ein überzogenes Konto kann nun in ein sogenanntes P-Konto umgewandelt werden, die Pfändungsfreigrenzen werden jährlich angepasst und statt einem Monat, kann künftig drei Monate auf dem Konto angespart werden.

Seit dem 1.10.2021 gelten nun neue, klarere gesetzliche Regeln für das Inkassokostenrecht. Sie werden begleitet vom Code of Conduct, einer Selbstverpflichtung der Inkassobranche, die aber kaum über die gesetzlichen Regelungen hinausgeht und an vielen Stellen sogar dahinter zurückbleibt.

Veränderungen gab es auch beim seit Jahren kontrovers diskutierten Thema der Restschuldversicherungen. Kritisiert werden hier seit Langem versteckte Kosten und mangelnde Kommunikation der Freiwilligkeit dieses Nebengeschäfts (unter anderem Reifner et al., 2013). Zum 1.7.2022 werden die Abschlussprovisionen bei Restschuldversicherungen nun auf 2,5 % des versicherten Darlehensbetrags begrenzt. Gerade für Personen mit geringen Einkommen sind faire und transparente Möglichkeiten für einen Kredit wichtig, denn Kredite können bei hohen Kosten zudem schnell zu Überschuldungstreibern werden. Die aufgezählten Themen betreffen zwar alle Bürger:innen gleichermaßen (und nicht nur überschuldete), die angesprochenen Änderungen haben aber eben gerade auf diese Einfluss, da sie sich häufiger in einer benachteiligten Position befinden.

Abbildung 1
**Überschuldete Personen in Deutschland**

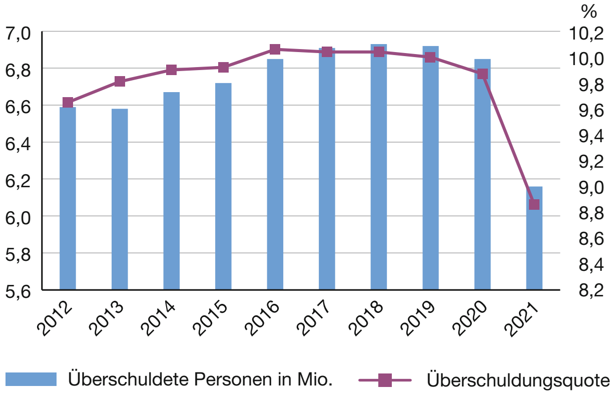

Quelle: Creditreform Schuldneratlas (2021, 61); Darstellung: iff.

## Anstieg Insolvenzzahlen massiv – aber erwartbar

Die Zahl der Verbraucherinsolvenzverfahren stieg im Jahr 2021 wie erwartet sprunghaft an. Besonders prägnant zeigte sich das im November 2021: Die Zahl der Verbraucherinsolvenzen im November 2021 verdreifachte sich fast gegenüber November 2020 (Destatis, 2022).

Es ist davon auszugehen, dass die Zahl der Insolvenzanträge sich wieder normalisieren wird, denn sie ist vor allem auf die Verkürzung des Insolvenzverfahrens von sechs auf drei Jahre zurückzuführen und nicht auf die Pandemie. Viele Ratsuchende haben mit Blick auf die Verkürzung des Insolvenzverfahrens ihren Antrag auf Eröffnung eines Insolvenzverfahrens zunächst zurückgehalten, denn Verbraucher:innen müssen (im Gegensatz zu Unternehmen) in der Regel nicht innerhalb einer bestimmten Frist einen Antrag auf Eröffnung des Verfahrens stellen. Die zeitweise geschlossenen Beratungsstellen und Gerichte haben diesen Antragsstau noch verschärft. Die Berater:innen mussten so zunächst einmal den Rückstand abarbeiten (Roggemann et al., 2021b, 30; Peters und Roggemann, 2021, 17 f.).

## Überschuldungsquote sinkt – ist doch alles gut?

Alljährlich veröffentlicht Creditreform die jährlichen Überschuldungszahlen (vgl. Abbildung 1). Die Veröffentlichung der aktuellen Zahlen im Oktober 2021 sorgte für großes Erstaunen: Die Zahl überschuldeter Verbraucher:innen ist im Oktober 2020 um fast 700.000 Fälle zurückgegangen und die Überschuldungsquote um mehr als einen Prozentpunkt auf 8,86 % gesunken (Creditreform Wirtschaftsforschung, 2021, 7). Das entspricht 3,08 Mio. Haushalten bzw. 6,16 Mio. Menschen (Creditreform Wirtschaftsforschung, 2021, 13). Die Quote hatte zwar auch schon die beiden Jahre zuvor abgenommen, allerdings nicht in einer Deutlichkeit wie im vergangenen Jahr. Es ist der niedrigste Wert seit Beginn der Auswertung im Jahr 2004 (Creditreform Wirtschaftsforschung, 2021, 61).

Der Rückgang betrifft mit 15,5 % insbesondere weiche Negativmerkmale, also z. B. Mahnungen oder die Beantragung eines Mahnbescheids. Ein Rückgang, der dreimal so hoch ist wie der Rückgang der harten Negativmerkmale mit 5,9 %, also gerichtlich bestätigter Umstände, wie Vermögensabgabe, Insolvenzverfahren (Creditreform Wirtschaftsforschung, 2021, 64). Der starke Rückgang der weichen Negativmerkmale ist insbesondere auf die umfangreichen Hilfspakete und die pandemiebedingten Beschränkungen zurückzuführen, die zu einem Ausgabeverzicht führten und so bei vielen Spielraum zur Rückzahlung der Schulden ermöglichten. Die Wirtschaft erholt sich trotz der nunmehr vierten Coronawelle und damit einhergehender weiterer Einschränkungen in einzelnen Bereichen zum Teil nur langsam. Weiterhin betroffen sind vor allem Handel und Gastronomie, zudem gibt es übliche saisonale Schwankungen.

Die Arbeitslosenquote lag im Januar 2022 bei 5,4 % (Bundesagentur für Arbeit, 2022, 16). Zum Vergleich: Das Vorkrisenniveau im März 2020 lag bei 5,3 % (Bundesagentur für Arbeit, 2022, 16). Die Folgen der COVID-19-Krise zeigen sich vor allem an einer verfestigten Arbeitslosigkeit bzw. an der gestiegenen Langzeitarbeitslosigkeit. Der Anteil der Langzeitarbeitslosen ist mit 30,3 % im März 2020 auf 40,2 % im Januar 2022 gestiegen (Bundesagentur für Arbeit, 2022, 13).

Vor dem Hintergrund, dass Arbeitslosigkeit in den vergangenen Jahren ein Hauptgrund für Überschuldung war und dies auch für 2020 und 2021 bestätigt wurde, ist zu befürchten, dass sich die Folgen der Coronapandemie in den kommenden Jahren in steigenden Überschuldungszahlen niederschlagen werden (Peters und Roggemann, 2021, 43). Leider ist (wie bei der Finanzkrise) daher davon auszugehen, dass sich die finanzielle Lage vieler Verbraucher:innen mit rund zwei Jahren Verzögerung auch in den Überschuldungsstatistiken zeigen wird. Das zeigte z. B. der iff-Überschuldungsreport 2009 nach der Finanzkrise (Knobloch et al., 2009, 26 ff.).

## Belastung für Verbraucher:innen und Beratungsstellen anhaltend hoch

Verschiedene Untersuchungen innerhalb der letzten zwei Jahre zeigen die schweren finanziellen Auswirkungen der Pandemie auf Verbraucher:innen (Roggemann et al., 2021b; Kaps et al., 2021). Die Situation hat sich immer nur phasenweise entspannt, bleibt aber insgesamt kritisch. Die negativen wirtschaftlichen Folgen der Pandemie verstärken auch die soziale Ungleichheit. Neben Frauen, die strukturell von Ungleichheit betroffen sind, sind auch Personen ohne Berufsausbildung eher finanziell von den COVID-19-Maßnahmen betroffen.

Auch die Ergebnisse einer repräsentativen deutschlandweiten Erhebung im Oktober 2020 zeigten bereits, dass die COVID-19-Pandemie voraussichtlich erheblich zu einer Erhöhung der privaten Überschuldung und zu einer Verschärfung der sozialen Ungleichheit führen wird. Eine finanzielle Überforderung vieler Menschen war bereits nach der ersten COVID-19-Welle nachweisbar und wird sich in Zukunft voraussichtlich für eine erhebliche Zahl an Menschen verschärfen (Roggemann et al., 2021b).

Entsprechend bedeutend ist der Zugang zu Unterstützungsleistungen zu bewerten. Doch die ohnehin an der Kapazitätsgrenze arbeitenden Beratungsstellen mussten sich in den vergangenen Jahren zusätzlichen Herausforderungen stellen. Die hohe Belastung der Schuldnerberatung bestätigen verschiedene Studien (Kaps et al., 2021, 23ff.; Roggemann et al., 2021b, 29 ff.). Übergreifend werden dabei von allen Studien vor allem die zunächst geschlossenen Beratungsstellen, fehlende Konzepte zur digitalen Überbrückung/Unterstützung, eine mangelhafte Finanzierung sowie der hohe Beratungsbedarf als Probleme genannt.

Im Rahmen des iff-Überschuldungsreports wurden teilnehmende Schuldnerberatungsstellen im Frühjahr 2021 gefragt, wie sich ihre Situation im Laufe der letzten 15 Monate entwickelt hat. Besonders prägnant waren dabei die folgenden Rückmeldungen: lange Wartezeiten, sehr hohe Arbeitsbelastung, zum Teil weiterhin nicht adäquate EDV, erschwerter Zugang zu Schuldnerberatungen (Peters und Roggemann, 2021, 29). Auch die Ergebnisse einer Umfrage der Arbeitsgemeinschaft Schuldnerberatung der Verbände (AGSBV) im Juli 2021 zeigen die anhaltende hohe Belastung: Bei über zwei Dritteln der befragten Beratungsstellen erhöhte sich die Zahl der Anfragen im Vergleich zum Zeitraum vor der Pandemie (AG SBV, 2022).

Finanzielle Auswirkungen der Pandemie zeigen sich zudem verzögert: Überschuldete kommen oft erst mit deutlicher Verzögerung in der Beratung an. Es vergehen durchschnittlich 3,5 Jahre vom ersten Ereignis, das im Zusammenhang mit der Überschuldung steht und dem Aufsuchen der Beratungsstelle, ein Viertel der Betroffenen braucht sogar 8,5 Jahre (Knobloch und Reifner, 2013, 74 f.). Bevor Menschen die Schuldnerberatung aufsuchen, werden alle anderen finanziellen Mittel ausgeschöpft. Es ist also mit einem weiterhin hohen Beratungsbedarf zu rechnen. Verbraucher:innen sind weiterhin hoch belastet; sie sind vor allem von steigenden Kosten in den Bereichen Wohnen, Energie, Lebenshaltung und Mobilität betroffen (unter anderem Destatis, 2022). Insbesondere Menschen mit knappem Einkommen oder gar schwerwiegenden finanziellen Problemen trifft das noch mehr. Weiterhin wird auch eine hohe Quote an Menschen vermutet, die keine Sozialleistungen in Anspruch nehmen. Das bringt Folgeprobleme mit sich. Nur wer im vergangenen Jahr in einem bestimmten Zeitraum Anspruch auf Leistungen wie Wohngeld hatte, bekommt z. B. nun auch einen Heizkostenzuschuss.

## Fazit

Vor allem aufgrund der Entwicklungen rund um COVID-19 erfuhr das Thema der privaten Ver- und Überschuldung viel Aufmerksamkeit. Eine erfreuliche Entwicklung war dabei, dass der Blick weg von der Individualisierung hin zu nicht planbaren Umständen, die eine Überschuldung verursachen können, gelenkt wurde. Die COVID-19-Pandemie hat dabei eine grundsätzliche Unterversorgung mit einer effizienten und effektiven Schuldnerberatung sichtbar gemacht. Schuldnerberater:innen und Überschuldete müssen die Konsequenzen tragen, dass Schuldnerberatung zum Teil mangelhaft aufgestellt war bzw. ist. Es gibt gute Ansätze, das grundsätzliche Problem der Überlastung bleibt aber vielerorts.

Die gesunkene Überschuldungsquote ist keineswegs ein Zeichen für Entspannung. Die Konsequenzen zeigen sich in der Regel erst in der Wirtschaft und dann bei den Verbraucher:innen. Die aktuellen Entwicklungen (vor allem in Hinblick auf die Energiepreise) erhöhen die Notwendigkeit einer erfolgreichen Schuldnerberatung weiter. Die derzeitige Lage ist allerdings weiterhin dadurch gekennzeichnet, dass es Ratsuchende nach wie vor sehr schwer haben, einen Zugang zu angemessenen Beratungsleistungen zu erhalten. Die Pandemie hat zahlreiche Defizite im Zugang und Angebot von Beratungsleistungen offenbart, die angesichts eines voraussichtlich steigenden Bedarfs an Beratung die Leistungsfähigkeit der Beratungseinrichtungen weiter infrage stellen könnten. Jetzt gilt es, die entsprechenden finanziellen Mittel zu mobilisieren und bei den überlasteten Strukturen Entlastung zu schaffen, um Menschen in finanziell schwierigen Situationen adäquate Unterstützung bieten zu können.

**Title:** ***Consumer Debt in Germany: Status and Development***

This article looks at over-indebtedness in Germany and its current status and trends. COVID-19 created increased attention on private debt and over-indebtedness. Various studies within the last two years show the severe financial impact of the pandemic; the situation has only eased in phases, but remains critical overall. A fundamental undersupply of efficient and effective debt counselling has become apparent. Current developments (especially with regard to energy prices) have increased the need for debt counselling.

Dr. Sally Peters ist Geschäftsführende Direktorin am institut für finanzdienstleistungen e. V. in Hamburg.


Literatur

AG SBV (Hg.) (2022), Umfrage der AG SBV: „Situation der Schuldnerberatung — Februar 2022“, https://www.infodienst-schuldnerberatung.de/umfrage-der-ag-sbv-situation-der-schuldnerberatung-februar-2022/(12. Februar 2022).
Bundesagentur für Arbeit (Hrsg.) (2022), Monatsbericht zum Arbeits- und Ausbildungsmarkt, Januar 2022, Berichte: Blickpunkt Arbeitsmarkt, https://www.arbeitsagentur.de/datei/arbeitsmarktbericht-januar-2022_ba147342.pdf (13. Februar 2022).
Bürgerbewegung Finanzwende (2020a), Dispozins runter! Zehn Prozent sind zu viel, https://www.flnanzwende.de/kampagnen/dispozins-runter/?L=0 (12. November 2020).
Bürgerbewegung Finanzwende (2020b), Wie viel Dispozins verlangt Ihre Bank? Deutschlandweiter Vergleich von mehr als 3400 privaten Girokonten, https://www.flnanzwende.de/themen/verbraucherschutz/dispozinsen/wie-viel-dispozins-verlangt-ihre-bank/?L=0 (24. Januar 2022).
Creditreform Wirtschaftsforschung (Hrsg.) (2021), SchuldnerAtlas Deutschland 2021. Überschuldung von Verbrauchern, https://www.boniversum.de/studien/schuldneratlas/ (11. Februar 2022).
Destatis (2022), 17,2 % weniger beantragte Regelinsolvenzen im Januar 2022 als im Vormonat, Pressemitteilung, Nr. 054, https://www.destatis.de/DE/Presse/Pressemitteilungen/2022/02/PD22_054_52411.html (13. Februar 2022).
Destatis (2022), Verbraucherpreisindizes. Verbraucherpreisindex: Sondergliederungen. Tabellen mit Jahresdurchschnitten und Monatswerten, Indizes und Veränderungsraten, https://www.destatis.de/DE/Themen/Wirtschaft/Preise/Verbraucherpreisindex/Tabellen/Verbraucherpreise-Sondergliederungen.html (20. Februar 2022).
Dick, C., M. Knobloch, K. S. Al-Umaray, L. Jaroszek, M. Schröder und A. Tiffe (2012), Studie zu Dispozinsen/Ratenkrediten. Forschungsvorhaben zur Bereitstellung wissenschaftlicher Entscheidungshilfe für das Bundesministerium für Ernährung, Landwirtschaft und Verbraucherschutz (BMELV).
Kaps, P., R. Reiter, F. Oschmiansky und S. Popp (2021), Wie sind soziale Dienstleister, ihre Mitarbeitenden und ihre Nutzenden von der Corona-Pandemie betroffen und wie wirken die sozialpolitischen Unterstützungsleistungen? Endbericht August 2021, ZEP — Zentrum für Evaluation und Politikberatung; Kaps & Oschmiansky Partnerschaftsgesellschaft von Politikwissenschaftlern, zep-partner.de/FIS_Coronafolgen_Dienstleister_ZEP_Endbericht_20210830.pdf (13. Februar 2022).
Klinger, H. (2021), Positionspapier: Entwurf der neuen Verbraucherkreditrichtlinie wird im Bundesrat beraten, https://www.iff-hamburg.de/wp-content/uploads/2021/09/PM-CCD_final.pdf (13. Februar 2022).
Knobloch, M., U. Reifner und W. Laatz (2009), iff-Überschuldungsreport 2009. Überschuldung in Deutschland, institut für finanzdienstleistungen e. V., https://www.iff-hamburg.de/wp-content/uploads/2019/01/iff_Ueberschuldungsreport_2009.pdf (17. Juli 2019).
Knobloch, M. und U. Reifner (2013), iff-Überschuldungsreport 2013. Unter Mitarbeit von Wilfried Laatz, Anna Nizkich und Christoph Wulf, https://www.iff-hamburg.de/wp-content/uploads/2019/01/iff_Ueber-schuldungsreport_2013.pdf (27. März 2020).
Lambrecht, C. (2021), (Video-)Grußwort der Bundesministerin der Justiz und für Verbraucherschutz. 18. Insolvenzrechtstag. Deutscher Anwaltverein, Arbeitsgemeinschaft Insolvenzrecht und Sanierung, 18. März, https://arge-insolvenzrecht.de/de/aktuelles/download-tagungsunterlagen (12. Februar 2022).
Peters, S. und H. Roggemann (2021), iff-Überschuldungsreport 2021, in Institut für Finanzdienstleistungen, 2021 https://www.iff-hamburg.de/2021/06/17/iff-ueberschuldungsreport-2021-pandemie-verschaerft-situation-fuer-ueberschuldete/ (22. Juni 2021).
Reifner, U., H. Klinger, M. Knobloch und A. Tiffe (2013), Fairness und Verantwortung im Konsumentenkredit — ein Bewertungsprojekt. Institut für Finanzdienstleistungen, https://www.iff-hamburg.de/wp-content/uploads/2013/12/Bericht_Fairness_20131118_FO1UR.pdf (12. Juli 2019).
Roggemann, H., H. Klinger, A. Fandrich, N. Korff, S. Peters, U. Reifner und I. Größl (2021a), Gutachten zum produktiven Kredit, Verbraucherzentrale Bundesverband (Hrsg.).
Roggemann, H., S. Peters und D. Korczak (2021b), Private Überschuldung in Deutschland. Auswirkungen der Corona-Pandemie und die Zukunft der Schuldnerberatung, Friedrich-Ebert-Stiftung (7).
